# Containing COVID-19 by Matching Messages on Social Distancing to Emergent Mindsets—The Case of North America

**DOI:** 10.3390/ijerph17218096

**Published:** 2020-11-03

**Authors:** Nick Bellissimo, Gillie Gabay, Attila Gere, Michaela Kucab, Howard Moskowitz

**Affiliations:** 1 School of Nutrition, Ryerson University, Toronto, ON M5B-2K3, Canada; michaela.kucab@ryerson.ca; 2 School of Behavioral Sciences and Psychology, College of Management Academic Studies, Rishon LeZion 91750, Israel; gillie.gabay@gmail.com; 3 Department of Postharvest and Sensory Evaluation, Institute of Food Technology, Faculty of Food Sciences, Szent Istvan University, H-1118 Budapest, Hungary; gereattilaphd@gmail.com (A.G.); mjihrm@gmail.com (H.M.); 4 MindCart AI, Inc., White Plains, NY 10605, USA

**Keywords:** communication, messages, compliance, COVID-19, health policy, social distancing

## Abstract

Public compliance with social distancing is key to containing COVID-19, yet there is a lack of knowledge on which communication ‘messages’ drive compliance. Respondents (224 Canadians and Americans) rated combinations of messages about compliance, systematically varied by an experimental design. Independent variables were perceived risk; the agent communicating the policy; specific social distancing practices; and methods to enforce compliance. Response patterns to each message suggest three mindset segments in each country reflecting how a person thinks. Two mindsets, the same in Canada and the US, were ‘tell me exactly what to do,’ and ‘pandemic onlookers.’ The third was ‘bow to authority’ in Canada, and ‘tell me how’ in the US. Each mindset showed different messages strongly driving compliance. To effectively use messaging about compliance, policy makers may assign any person or group in the population to the appropriate mindset segment by using a Personal Viewpoint Identifier that we developed.

## 1. Introduction

Scientists and policy makers have been concerned about the emergence of an influenza pandemic for which there is neither a strain-specific vaccine nor sufficient antiviral medications [1]. Social distancing is effective in reducing infection rates within communities [2,3,4,5]. Measures of social distancing undertaken in Italy, Spain, India, and most of the United States are drastic [6]. Local governments across the world impose four practices of social distancing including isolation of people with probable influenza; voluntary home quarantine for people with confirmed or probable influenza; preventing the congregation of people in community or employment settings; and social distancing of adults [4,7,8].

The implementation of social distancing requires significant public cooperation [4]. Previously, compliance was high in the early stages of the pandemic but decreased significantly over the course of several months [9]. Further, the compliance of people belonging to racial and ethnic minorities and to low socio-economic groups has been low [10,11]. Practical, logistical and ethical limitations of social distancing have also affected compliance [5]. Compliance with the policy of social distancing may maintain the resilience of communities. Designing communication interventions may reduce the burden of the psychological and social consequences of social distancing and reduce trauma that may transcend generations, constraining coping capacities [12]. In previous pandemics, people with low tolerability to policies fled from infected cities, participated in riots, attacked government officials and harmed those suspected of plaguing [1]. Compliance with social distancing may also be influenced by one’s level of perceived risk, one’s perception of individual and family disruption, and the perceived effectiveness of agencies dealing with the crisis [13,14,15]. The resilience of communities may depend on the extent of compliance with social distancing maintained in those communities. The maintenance of compliance will depend on appropriate communication [16]. Thus, communication-/messaging regarding social distancing, must consider reactions to the policy, and avoid the creation of resistance, which can endanger compliance [17]. The purpose of this study is to evaluate the specific communication messages that have the potential to act as drivers of compliance to social distancing.

Most studies on public behaviors in a pandemic tested the extent of communication that the public needs for compliance [16]. Research on the impact of specific communication messages on compliance is scant [17]. Previous studies called to close the gap and examine how to persuade the public to comply with social distancing [15,18,19]. Studies also suggested using controlled research designs rather than observational studies [20]. This study responds to previous calls seeking to discover communication messages that are likely to resonate positively with the individual, thus enhancing compliance. Higher compliance with social distancing will allow hospitals to manage their overflow, until a vaccination is available [1]. Research also acknowledged that a strategy using various communication messages to optimize compliance with social distancing may be more effective than using one message for all [21]. Furthermore, during pandemics, the communication resources of governments become scarce. Effective messaging enables policy makers to allocate resources based on real, immediate, and relevant data.

Effective health communication encompasses narrative messaging [22,23]. Narrative messages may be effective in the context of pandemic behavior as well, because they are the basic mode of interaction that people use to influence others [24,25,26,27]. The impact of messages may be explored by asking questions about whom, under what circumstances, how, and when does each message achieve an optimum effect [28]. The power of specific messages may vary across individuals, possibly as a consequence of their perceived risk. Furthermore, the effect of narrative messaging on compliance to social distancing may depend, in part, on the extent to which people “identify” with the message [28]. People may, therefore, be defined by the similarity in their pattern of responses to narrative persuasion messages (i.e., mindset segments) regarding social distancing. Therefore, we hypothesized that various messages will be strong drivers of compliance, while others will be weak drivers. We further hypothesize that the power of the messages as drivers of social distancing will differ based on the similarity in patterns of response to the messages.

## 2. Materials and Methods

Ethics: This study protocol was approved by the Ryerson University Research Ethics Board (#2020-149).

### 2.1. Study Sample

Respondents were comprised of 106 Canadians and 118 Americans—100 males and 124 females, who were 18–77 years of age (Table 1). This sample size is consistent with the suggested sample size in conjoint analysis studies, particularly when aiming at stability of coefficients rather than stability of means [29]. The recruiting service (Luc.id, Inc., New Orleans, LA, USA) invited the respondents, incentivized them, and rerouted the respondent to a Mind Genomics^®^ online study on compliance to social distancing during a pandemic.

### 2.2. Procedure and Instrument

The dependent variable is social distancing rated on a 1–5 anchored scale (1 = disagree, 5 = agree). The independent variables were four categories of messages representing known drivers of compliance [13,14,15]. Narrative messages tested in this study are drivers of the intention to comply, which were found to strongly relate to the behavior of compliance [30]. The independent variables result in sixteen messages that were randomly mixed into 24 combinations. The approach varies the combinations by a permutation scheme [31]. Each respondent rated a unique set of 24 combinations of messages comprised of 2–4 variables. Respondents were instructed to rate each combination of messages as a whole [32,33,34,35]. Messages may be incoherent, pulling in different directions [35,36,37]. Underlying the combinations is a well-crafted experimental design [35,36]. The uniqueness of combinations is ensured by the permutation scheme, which keeps the structure of the 24 combinations the same from one respondent to another but varies the specific combinations [38]. The Mind Genomics^®^ approach parallels complexity in the real world, where people are presented with compound information from different sources and must make rapid decisions. The response to these combinations, uncovered by ordinary least-squares regression (OLS), reveals the importance that each respondent attributes to each message, with the judgment bias reduced [38,39]. Table 2 presents the study instrument.

### 2.3. Statistical Analysis

We used conjoint analysis, a statistical procedure, to determine the messages that drive social distancing. The experimental design enabled the deconstruction of responses to the messages by OLS [34,35]. We created models for willingness to comply using OLS, one for each respondent, each with an additive constant and 16 coefficients (i.e., one coefficient for each message). The additive constant is an estimated parameter representing the intercept in a linear equation that may be interpreted as the predisposition of the respondent group to agree to a set of messages in the absence of any specific message.

To highlight the best-performing messages and eliminate a high variability due to lack of calibration among respondents, we transformed the ratings to a binary scale. Ratings 4 and 5 (upper 40% of the scale) were transformed to 100, classified as positive outcomes (‘Top2′); and ratings below 4 (lower 60% of the scale) were transformed to 0, classified as negative outcomes. After the transformation, a small random number between 0.1 and 0.01 was added to make the variable continuous. OLS analysis was performed to create an individual-level regression model for each respondent. This type of individual regression approach has been widely used in conjoint analysis studies [40]. The OLS model was written as follows:

(1) $$\hat{Y}=\beta_{0}+\beta_{1}X_{1}+\beta_{2}X_{2}+\cdots +\beta_{p}X_{p}$$

where $\hat{Y}$ is the predicted or expected value of the dependent variable (here, the transformed, binarized ratings),$\;X_{1}$ through $X_{p}$ are p distinct independent or predictor variables, $\beta_{0}$ is the value of Y when all of the independent variables ($X_{1}$ through $X_{p}$) are equal to zero, and $\beta_{1}$ through $\beta_{p}$ are the estimated regression coefficients.

The OLS coefficient is the conditional probability that the specific message adds to the perceived importance of the additive constant for social distancing. OLS was run for the entire panel, incorporating all relevant data into one regression model for the sample. The regression model, estimated at the level of each respondent, is appropriate because of the permuted design and allowed clustering based upon the 16 coefficients. *k*-means clustering was applied on the 16 coefficients to create clusters. These clusters represent mindsets because they suggest what is important to the respondent for the specific topic. Mindsets emerge from the pattern responses to the specific, relevant messages, not from stated attitudes. Following mathematical clustering, the equation for each key subgroup was estimated using all data from the appropriate group. Analysis of variance and post hoc tests indicate whether the distinct mindset models were significant, highlighting the different messages that drive social distancing for each mindset. The pattern of positive high coefficients across different mindsets guided the assignment of respondents to mindset.

## 3. Results

### 3.1. Interpretation

To simplify the analysis, we present only messages with positive coefficients, driving agreement with compliance. Negative coefficients mean either that the element is neutral (irrelevant for compliance) or counterproductive, driving non-compliance. Table 3 shows messages that drive compliance among Canadians. Table 4 shows messages that drive compliance among Americans. Strong drivers of compliance with social distancing for the total sample were “To practice social distancing, everyone should stay six feet apart from others” and “The media or central government should communicate the social distancing policy”. Respondents perceived the government as reactive rather than proactive, failing to understand the significance of COVID-19.

The additive constant shows the estimated, baseline likelihood that a respondent ‘agrees’ with the message. The data show that the readiness to comply is higher among Canadians than Americans, as shown in Table 3 and Table 4.

### 3.2. Mindset Segments

Three mindsets emerged from mathematical K-clustering based upon similarity in the patterns of responses to the individual messages [41]. The mindsets transcend the typical patterns used to divide respondents, namely WHO they are, summarizing how people THINK, or the nature of what people feel about social distancing.

Mindsets 1 (MS1) and 2 (MS2) are similar in both countries. People belonging to MS1, “Tell me exactly what to do”, may be driven to higher compliance by messages regarding ways to ensure compliance. Strong messages were “*To ensure compliance, people should be under military lockdown*”, “*Compliance may be ensured by restricting shopping for food (3 at a time) and pharmacy (1 at a time)”*, and “*Designated young volunteers should shop for the elderly and disabled*”. People belonging to MS2, “Pandemic onlookers”, may be driven to higher compliance by messages relating to the perceived risk of the virus. Strong messages were “*A dangerous virus is spreading wildly*”, “*All the news seems to be about the COVID-19 virus*”, and “*Health experts suggest…government is reactive rather than proactive to the pandemic*”. People belonging to this mindset may agree with any social distancing practice.

Mindset 3 (MS3) differs dramatically between countries. Canadian respondents appear to respond to authority, whereas American respondents appear not to respond to authority. Canadians belonging to MS3, “Bow to authority”, are driven to higher compliance by the communicator of the policy and believe that either provincial government, federal government, the media or religious clergy should communicate the social distancing policies. Americans belonging to MS3, “Tell me how”, may be driven to higher compliance by messages that focus on the practices of social distancing. Strong messages were *“Everyone stays 6 feet apart”, “Socialize and work only from home on the internet”, “Confined to within 300 feet from home”, and “Wear your mask everywhere”.*

### 3.3. Personal Viewpoint Identifier

For effective knowledge translation, the emergence of these findings requires an innovative way to assign a new person or group in the population to these previously discovered mindsets. To translate the knowledge derived in this study for implementation, we developed a prediction tool called a Personal Viewpoint Identifier (PVI). The PVI tool is a method by which health authorities may assign a person or group in the population to a mindset. This is based on the summary data, converting six of the strong distinguishing messages to questions answered by a “agree or disagree”. The six messages were chosen using a Monte-Carlo simulation where random variability is added to the data, and the most discriminating messages emerge in the face of random variability [42]. Based on the answers to the questions in the PVI, the individual or group is assigned to one of the three mindsets, and thus, the proper messages are established for that individual (Table 5).

## 4. Discussion

This study examined the influence of numerous communication messages on compliance with social distancing in North America. Communication messages represent relevant, topic-specific ideas. Ordinarily, one would tailor the messaging to who a person IS demographically. This study suggests tailoring messaging by the way the person THINKS about compliance to a health policy in a pandemic, particularly because messaging shaping public behavior during a pandemic is an undertested phenomenon. This study revealed messages that drive positive responses to social distancing, varying by individuals or groups that think alike. The first hypothesis was corroborated as some messages were strong drivers of compliance and some were weak drivers of compliance. Results echo previous research claiming that the extent to which messages drive social distancing may depend, in part, on the extent to which people “identify” with the different narrative messages [28].

The second hypothesis was also corroborated as three mindsets emerged from the similarities in patterns of responses to narrative communication messaging regarding social distancing. Even during the midst of a pandemic, when social distancing is critical and novel, approaching each of the three different mindset segments with appropriate communication messages should enhance compliance with social distancing. Health policy makers and government leaders should take into consideration the proclivities and sensitivities of these three mindsets when suggesting how to properly implement the policy of social distancing over time.

Findings of this study should be interpreted in light of its limitations. Our study variables reflect our choice of constructs, which was based on prior literature, but does not encompass additional independent variables that are not yet acknowledged as potential drivers of compliance with social distancing. Further, given the continuing pandemic, respondents may have been previously exposed to messages on social distancing in real life before participating in this study. The exposure to messages, the frequency of the exposure and the potential prime effect of messages may have influenced the extent of agreement with messages regarding social distancing. These external cofounders, however, cannot be controlled in a cross-sectional design.

### Directions for Future Studies

Future studies may track the relationship between using communication messages by mindset segment membership and compliance with social distancing as measured by cellular data. Longitudinal future studies may also test the prime effect of communication messages and the effect of exposure to messages and their frequency on the level of compliance.

## 5. Conclusions

The use of the Personal Viewpoint Identifier for enhancing public compliance with social distancing may be a non-intrusive way to understand the individual. This allows us to communicate a message with greater specificity and have a better chance of driving compliance with physical/social distancing, highlighting the potential collaboration between academia and health officials to contain COVID-19 [43].

## Figures and Tables

**Table 1:** Participant characteristics.

Variable	Canada	United States of America
Gender (Male/Female)	52/54	48/70
Age (Mean ± SD)	45.6 ± 15.1	48.3 ± 15.6

**Table 2:** The four independent variable categories, and the four test messages to be incorporated into the vignette.

Code	Elements
	Independent Variable A: The Perceived Risk of COVID-19
A1	A dangerous virus spreading wildly
A2	Media have overblown new strain of influenza… people panicking
A3	All news seems to be about the COVID-19 virus
A4	Health experts suggest… but government is reactive rather than proactive to pandemic
	Independent Variable B: Practices of Social Distancing
B1	Socialize… work only from home through internet, e.g., Zoom/Skype
B2	Everyone stays 6 feet (2 m) apart
B3	Confinement to within 300 feet (100 m) from home
B4	Wear your mask everywhere
	Independent Variable C: Ways to Ensure Compliance to Social Distancing
C1	Military lockdown
C2	Food shopping (3 people at a time) … pharmacy (1 person at a time) … gas (attendant dispenses)
C3	Designated young volunteer for priority shopping… for elderly and disabled
C4	Only age 60+ allowed to buy groceries during first 2 h of store day
	Independent Variable D: The Agent Communicating the Policy
D1	State or provincial government should communicate the policy
D2	Federal government should communicate the policy
D3	Religious clergy should communicate the policy
D4	The media should communicate the policy

**Table 3:** Three mindset segments (MS) for Canada.

	MS1: Tell Me Exactly What to Do	MS2: Pandemic Onlookers	MS3: Bow to Authority
Sample Size	28	39	39
Additive constant (agree, without any messages)	60	41	41
Independent A—The perceived risk of COVID-19			
A1: Dangerous virus spreading wildly		17 *	
A2: Media have overblown new strain of influenza… people panicking		14 *	
A3: All news seems to be about the COVID-19 virus		12 *	
A4: Health experts suggest…government is reactive rather than proactive to pandemic		11 *	
Independent B—Practices of social distancing			
B1: Socialize… work only from home on internet, e.g., Zoom/Skype			3
B2: Everyone stays 6 feet (2 m) apart	2		1
B3: Confined to within 300 feet (100 m) from home			1
B4: Wear your mask everywhere			6
Independent C—Ways to ensure compliance to social distancing			
C1: Military lockdown	15 *	0	
C2: Food shopping (3 people at a time) … pharmacy (1 person at a time) … gas (attendant dispenses)	17 *		
C3: Designated young volunteer for priority shopping… for elderly and disabled	21 *		2
C4: Only age 60+ allowed to buy groceries during first 2 h of store day	6		
Independent D—The agent communicating the policy			
D1: Provincial government should communicate the policy			14
D2: Federal government should communicate the policy			13
D3: Religious clergy should communicate the policy			19 *
D4: The media should communicate the policy			13

Regression coefficients for models relating the presence/absence of the elements to the rating of disagree/agree, after binary transformation. * denotes significant, positive model parameters (*p* < 0.05).

**Table 4:** Three mindset segments (MS) for the United States of America.

	MS1: Tell Me Exactly What to Do	MS2: Pandemic Onlookers	MS3: Tell Me How
Sample Size	29	43	46
Additive constant (agree, without any messages)	43	52	40
Independent A—The perceived risk of COVID-19			
A1: Dangerous virus spreading wildly	4	7	
A2: Media have overblown new strain of influenza… people panicking		13 *	2
A3: All news seems to be about the COVID-19 virus		13 *	
A4: Health experts suggest…government is reactive rather than proactive to pandemic	0	6	
Independent B—Practices of social distancing			
B1: Socialize… work only from home on internet, e.g., Zoom/Skype			10 *
B2: Everyone stays 6 feet (2 m) apart			9 *
B3: Confined to within 300 feet (100 m) from home			14 *
B4: Wear your mask everywhere			14 *
Independent C—Ways to ensure compliance to social distancing			
C1: Military lockdown	15 *		
C2: Food shopping (3 people at a time) … pharmacy (1 person at a time) … gas (attendant dispenses)	6	1	
C3: Designated young volunteer for priority shopping… for elderly and disabled	14 *	2	
C4: Only age 60+ allowed to buy groceries during first 2 h of store day	9 *	2	
Independent D—The agent communicating the policy			
D1: State government should communicate the policy			2
D2: Federal government should communicate the policy			3
D3: Religious clergy should communicate the policy	3		1
D4: The media should communicate the policy	1		5

Regression coefficients for models relating the presence/absence of the elements to the rating of disagree/agree, after binary transformation. * denotes significant, positive model parameters (*p* < 0.05).

**Table 5:** Personal Viewpoint Identifier.

DRIVERS OF SOCIAL DISTANCING PVI	
Question 1: How do you feel about COVID-19?	○ Under control now
	○ Under control soon
	○ A lot worse than we think
	○ I have no idea
Question 2: What do you fear most right now about COVID-19?	○ Medical system breakdown
	○ Economic system breakdown (jobs, livelihood)
	○ Education system breakdown
	○ Social interaction system breakdown
Question 3: Who do you trust most?	○ Clergy
	○ Government
	○ News
	○ Group of friends
Question 4: How long do you think COVID-19 will last?	○ About 2 months
	○ About 4 months
	○ About 2 waves of 2–3 months
	○ I have no idea
Question 5: Situation: Media have overblown new strain of influenza… people panicking	○ Agree
	○ Disagree
Question 6: Situation: Dangerous virus spreading wildly	○ Agree
	○ Disagree
Question 7: Compliance Policy: Food shopping (3 people at a time) … pharmacy (1 person at a time) … gas (attendant dispenses)	○ Agree
	○ Disagree
Question 8: Who Communicates: Religious clergy	○ Agree
	○ Disagree
Question 9: Who Communicates: Provincial government	○ Agree
	○ Disagree
Question 10: Compliance Policy: Designated young volunteer for priority shopping… for elderly and disabled	○ Agree
	○ Disagree
